# A Sleeve-Sheath With a Coaxial Exchangeable Catheter for Palliative Evacuation of Recurrent Thoracic and Abdominal Effusions

**DOI:** 10.7759/cureus.25174

**Published:** 2022-05-20

**Authors:** Kevin Malone, Christopher M Stevens, Chandler Merriman, Daniel Harper, Reena Wadhwa, Horacio R D'agostino

**Affiliations:** 1 Biomedical Engineering, Louisiana State University Health Sciences Center, Shreveport, USA; 2 Interventional Radiology, Louisiana State University Health Sciences Center, Shreveport, USA; 3 Radiology, Louisiana State University Health Sciences Center, Shreveport, USA

**Keywords:** interventional radiology, cannula sleeve-sheath, exchangeable drainage catheter, hospice and palliative care, malignant pleural effusions

## Abstract

Herein we describe an outer cannula sleeve-sheath with a coaxially inserted exchangeable drainage catheter (SCDC) for effective evacuation of recurrent symptomatic fluid collections in the thorax and abdomen on patients in lieu of, or failed, current evacuation catheters and methods. The design is an alternative to existing commercially available devices and adds distinct enhancements with the possibility of intrathoracic or intrabdominal trans outer sleeve-sheath diagnostic or therapeutic interventions. This device aims at requiring a single invasive procedure (thoracentesis and paracentesis) while offering catheter exchange and repositioning if malfunction or malposition occurs during the patient’s lifetime. The SCDC outer sheath in the subcutaneous tissues of the thorax or abdomen has built-in two antibacterial cuffs to prevent infection. At the same time, the exchangeable coaxially inserted drainage catheter is deployed over a guidewire within the thoracic or abdominal cavities. The drainage catheter has a fluid dynamic proven efficient design to facilitate drainage and can recanalize its lumen if occluded by fibrin or tissue.

## Introduction

Malignant effusions are a common occurrence in palliative care patients and often cause significant discomfort [1]. According to the World Health Organization, approximately 40 million people require palliative care each year [2]. In 2018, there were over 1.55 million persons terminally ill under hospice care, of whom 29.6 percent had a leading diagnosis of cancer [3]. Common sites for effusions requiring repeated drainage include the pleural space, pericardial space, and the abdominal cavity. When effusions become symptomatic, evacuation of the fluid is required. Typically, the effusion removal involves a temporary device placed under local anesthetic or generalized anesthesia. For pleural effusions, a chest tube is usually inserted posteriorly between the ribs just directly above the ninth or tenth rib to remove fluid [4]. Conversely, symptomatic ascites is evacuated through paracentesis. The procedure typically involves the insertion of a needle, usually in the left or right lower quadrant of the abdomen, to remove the fluid. Both involve local anesthesia of the soft tissues (eventually, sometimes general anesthesia is required in noncooperative or pediatric patients) [5]. These procedures, while generally well tolerated, can be uncomfortable. If the underlying cause is not corrected, patients in palliative care may frequently have a recurrence of symptomatic pleural or abdominal effusions, impairing their quality of life [6]. Evacuation of these effusions entails repeated visits to the hospital, with inherent periprocedural laboratory tests and trauma to the patient.

The SCDC device, with its outer cannula sleeve-sheath and coaxially inserted exchangeable drainage catheter, aims at simplifying and effectively drain recurrent effusions, reduce the number of uncomfortable and invasive procedures, and alleviate discomfort. The device would require a single invasive procedure and will remain within the patient allowing for easy access and drainage. Special considerations were implemented to reduce the possibility of infection with the development of the device and with mechanisms allowing for catheter exchange with technology that is currently commercially available and approved by the FDA.

## Technical report

Defining the problem and solution

The current problem is that there are suboptimal solutions to minimally invasive long-term drainage of both pleural fluid and ascitic fluid for palliative patients. Standard methods of care for recurrent effusions include thoracentesis, paracentesis, pleurodesis, the use of PleurX, and other similar catheters. However, they all have drawbacks and are not ideal methods of treatment (Table 1). In more recent years, new devices were developed that intend to reduce the pain and impaired quality of life by decreasing the number of invasive procedures [7]. Unfortunately, current market solutions are not without complications, such as infections, blockages, and device failure. Given these circumstances, there is a need to improve on this idea and even expand on it.

**Table 1:** Current Problems in Procedural Management of Recurrent Symptomatic Thoracic and Abdominal Effusions

Procedure Type	Problem with Procedure
Repeated thoracentesis	Multiple invasive procedures may become intolerable for inpatients and burdensome for outpatients while exposing them to complications such as infections, pneumothorax (seen in 6% of patients), and hemothorax (seen in 2% of patients) [8].
Repeated paracentesis	Multiple accesses to the abdomen can be complicated by bowel perforation, bacterial peritonitis (seen in 2.62% of patients), and hemorrhage (seen in 2.87% of patients) from paracentesis [9].
Pleurodesis with talc slurry or doxycycline	A means of controlling recurrent effusions with an effectiveness of 81.8% was seen at six weeks. However, the effectiveness decreased to 60% and 21.8% at six and 12 months, respectively. The failure rate was initially 18.2% at six weeks but subsequently increased to 40% at six months and 78.2% at 12 months [10].
PleurX^TM^ Catheter	Catheter insertion has an effectiveness of 86%, failure of 14%, and complication rate of 7% which included pneumothorax and infection. However, PleurX catheters were only able to remain functional for less than a year [11,12].
Peritoneovenous Shunt	A peritoneovenous shunt acts as a connection between the systemic venous circulation and the peritoneal cavity. It may become infected or become blocked.

The primary goal of the SCDC is to help alleviate the burden of patients with recurrent pleural and abdominal effusions; however, the device can also aid physicians by decreasing the amount of work capacity for physicians and other healthcare providers. Physician burnout rates reached as high as 42% in recent years, which results in reduced productivity and efficiency of care to patients [13]. Reduction in physician stress and workload can improve the quality of care their patients receive, leading to better outcomes. The ways in which this device would help both patients and physicians include a decrease in patient hospital visits, decreased number of invasive procedures, reduced hospital costs, and reduced workload for physicians; this would then help with the current physician shortage and physician burnout, and still provide for a better quality of care for patients due to reduced workload for physicians. In addition, the ability to exchange the coaxial drainage catheter through the outer sheath with minimal complexity and trauma to the patient is a robust desirable advantage above the products on the market.

## Discussion

Design

**Figure 1:**
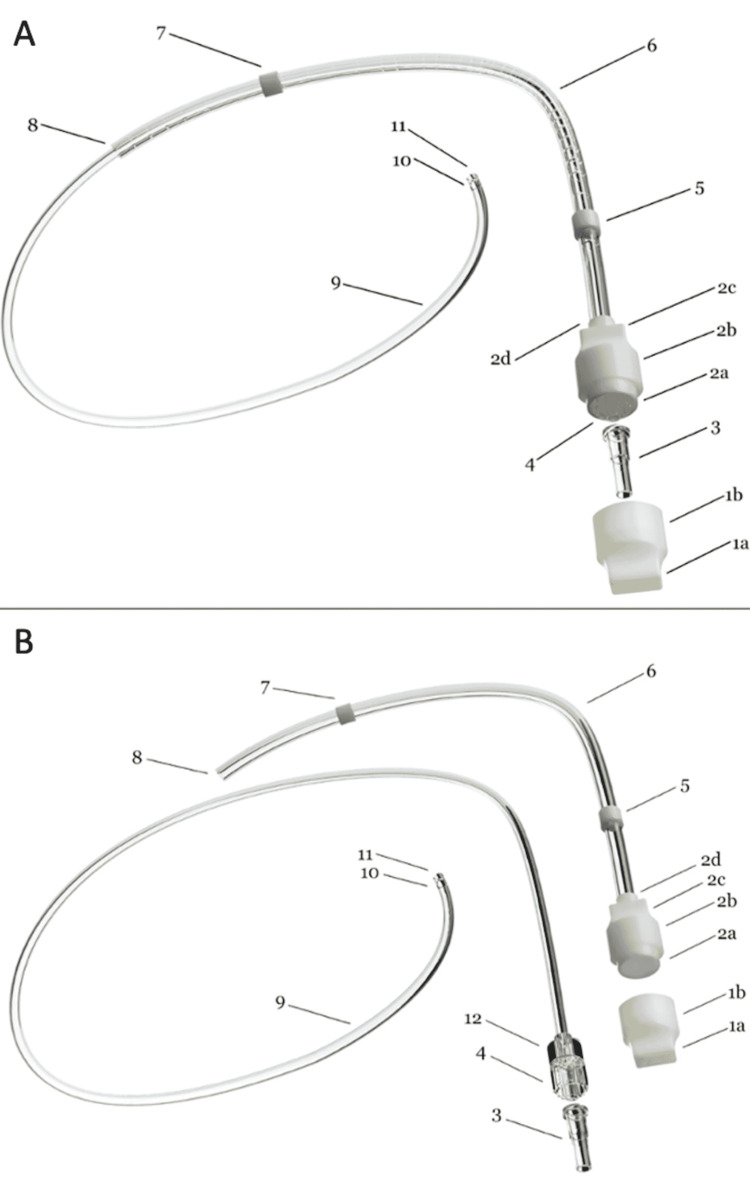
The SCDC and components with the Coaxial Exchangeable Catheter traversing the sleeve-sheath (A) and the Coaxial Exchangeable Catheter separate from the sleeve-sheath (B). 1a. Tip of end-cap that is shaped for ergonomic easy removal and reduction of size. 1b. The body of the end-cap, which is oval-shaped for easy twisting motion. 2a. Lip of main-cap which fits inside of end-cap for sterile connection. 2b. The body of the main-cap. 2c. The main-cap has a tapered reduction to reduce size. 2d. Extra length of the main-cap to allow for stability of connection to the outer sheath tube (permanent connection point). 3. Luer-lock end-cap which should remain sterile and to close the inner catheter. 4. Luer-lock connection which is permanently connected to the inner catheter. 5. Proximal antimicrobial microfiber. 6. Distal antimicrobial microfiber. 7. The outer sheath tube with a removable inner catheter running inside. 8. Distal end hole of outer sheath which would be just outside the pleura. 9. Inner removable and exchangeable catheter. 10. One proximal end hole on the inner removable and exchangeable catheter. 11. One distal hole of the removable inner catheter. 12. Inner removable catheter that allows for simple manipulation.

**Figure 2:**
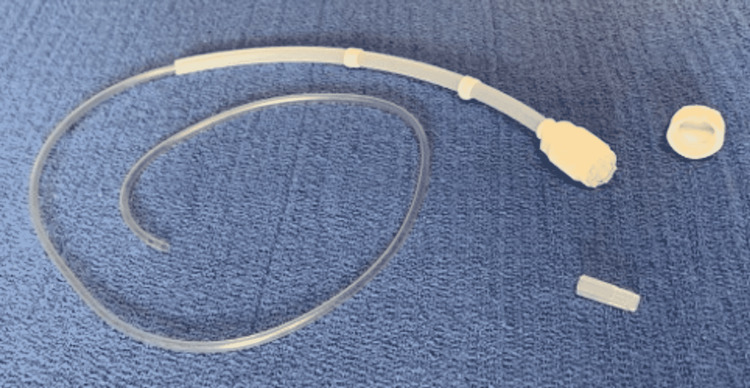
Current prototype with coaxial exchangeable catheter residing within the sleeve-sheath.

**Figure 3:**
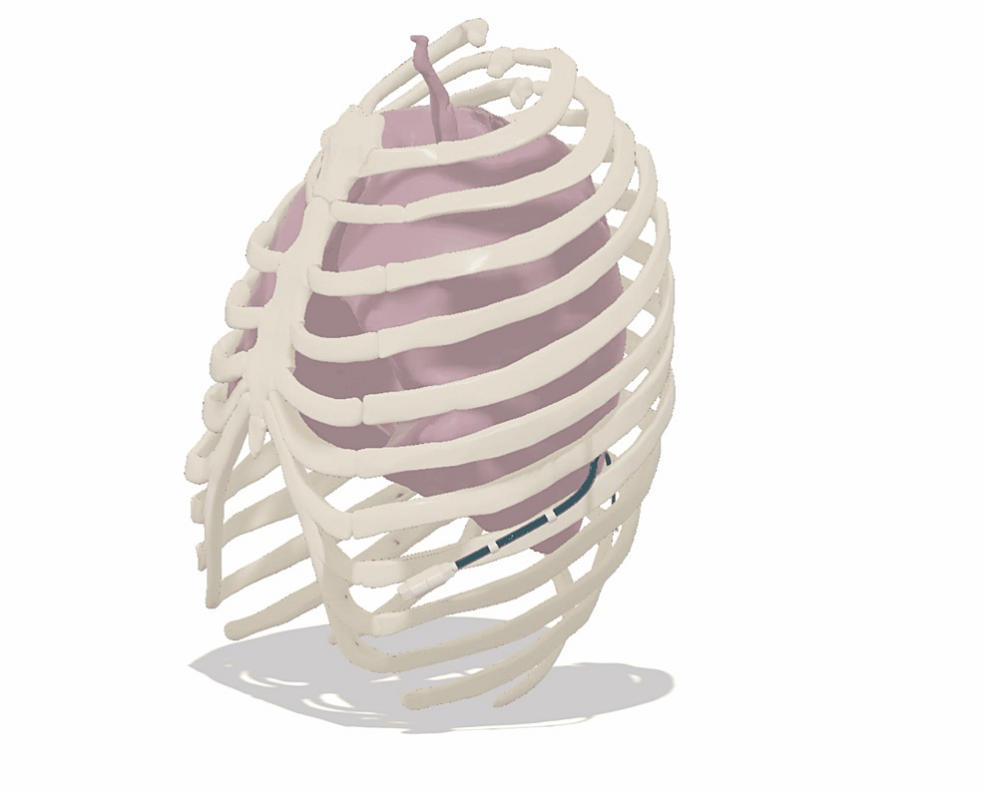
3D model of the current prototype as it would reside within the chest.

The SCDC (Figures 1-3) was intended explicitly for being able to perform efficiently while preventing infection. The hub system has a shell of plastic, which is capped, and an inner sterile compartment. The inner compartment gives access to both the sheath and the inner coaxial catheter. The 12-14 F, 10 cm length, outer sleeve-sheath has two antimicrobial cuffs for infection prevention. The 10-12 F, 40-60 cm coaxial inner catheter was specifically designed following fluid dynamics for effective fluid evacuation. The outer sleeve-sheath remains in the subcutaneous tunnel without entering the pleura or traversing the parietal peritoneum. The coaxial inner catheter enters the pleural or abdominal cavities. The catheter tip is to be deployed in the location selected by imaging for better drainage.

The coaxial inner catheter is a straightforward and inexpensive design. The catheter tip has a single inner lumen diameter side hole near the blunt tip, which would allow for excellent drainage. Studies have shown that just one side hole proximal to the end hole provides optimal drainage [14]. According to recent studies, unilateral model catheters, with more than three side holes, did not have a significant change in drainage volume [14,15]. Any additional holes that are not draining adequately can cause stagnation where clots and other thick material can clog the tube and render the catheter ineffective. This coaxial inner catheter tip design allows for easy permeabilization of the lumen by saline or alteplase solution syringe flushing.

Thoracic and abdominal effusions, with rare exceptions, tend to be of lower viscosity. Therefore, 10-12 F diameter of the coaxial drainage catheter will suffice for effective evacuation. Coaxial inner catheter exchange might be done regularly or scheduled to avoid inner lumen fibrin buildup with a simple sterile procedure. In the event of catheter malfunction or malposition, the outer sleeve-sheath allows for removal of the coaxial inner catheter and exchange over a guidewire. Malposition may be corrected with wire manipulations or removal of the catheter over a guidewire and insertion of a guiding catheter. Once the desired positioning of the guidewire is achieved, the coaxial catheter is inserted. Additionally, the outer sleeve-sheath allows for intrathoracic and intra-abdominal diagnostic and therapeutic manipulations. The SCDC is ready for the most important and upcoming practice of using mini endoscopy (SpyGlassTM). The thoracic and abdominal cavities could be examined with videoendoscopy. Such method allows for selective controlled tissue sampling (biopsy), lysis of fibrin septa loculations that may develop within the effusions, and tissue ablation.

The skin sleeve-sheath skin entry site location is to be located for visual inspection, frequent cleaning, and application of a Chlorhexidine impregnated Tegaderm. The external securement device would likely be sutured in place for maximal securement or secured with an external fixation device. Fluid evacuation may be achieved with a connecting tube from the Luer-lock to a vacuum container or a collection container attached to low wall suction.

Considerations for the size of the sheath and catheter were made. The catheter needs to be small enough to allow for easy manipulation while in the sheath, but also large enough to stop the sheath from being displaced due to drag during drainage. Due to the potential solution preventing fibrin buildup and maximization of patient comfort, it was proposed that a smaller French catheter and sheath would be possible. Current models are based on 12-14 French sheaths and 10-12 French inner catheters; however, this would need to be observed clinically to determine if these sizes are reasonable long-term solutions. Both the sheath and the catheter would likely be made from polyurethane which is a commercially available industry standard.

SCDC placement

The Interventional Radiology suite is the best procedure room where the SCDC is to be placed under sterile technique with ultrasound (US) and fluoroscopy guidance. The patient’s clinical condition and available resources may limit access to fluoroscopy. In such circumstances, the procedure may be performed at the patient’s bedside or in a room where an ultrasound is available for guidance.

SCDC Placement for Recurrent Pleural Effusions

In planning the SCDC skin exit site, considerations for long-term patient comfort, easy access and visual inspection of the skin exit site to check for redness or exudate are important. Therefore, the skin exit site should try to be in the anterior or midaxillary line. Sonographic imaging will identify the pleural effusion and for standard pleural access with the needle entry on top of the rib, guidewire insertion, and coiled within the pleural effusion space. Subsequently, a subcutaneous tunnel from the pleural access site to the anterior or midaxillary axillary line is created. The subcutaneous tunnel travels along the rib to prevent damage to the neurovascular bundle. The outer sheath skin exit site is clearly visible and both antimicrobial cuffs are to remain within the subcutaneous tunnel. These antimicrobial cuffs have been described in the literature as binding to subcutaneous tissue and helping to retain the catheter in place [16]. The inner cannula would then transverse the sleeve-sheath and reside within the pleura.

SCDC Placement for Recurrent Abdominal Effusions

The location of the sleeve-sheath exit site may vary. Similar considerations to the SCDC skin exit site in the thorax are applied: easy access and visual inspection, and patient comfort. Additional SCDC placement steps are standard keeping the outer sheath-cannula within the subcutaneous tissues of the abdominal wall or lower rib cage, and the inner coaxial catheter tip placed in the dependent portion of the larger pocket of intraabdominal fluid.

SCDC Catheter Exchange and Reposition

In the event of catheter malfunction (occlusion, fibrin sheath), malposition by imaging, or electively after 1-2 months, the coaxial drainage catheter can be exchanged over a guidewire. Reposition may be done at the time the catheter malposition is identified as causing malfunction. Under sterile conditions, the inner catheter is accessed. A hydrophilic stiff guidewire is used to remove the existing coaxial catheter. If needed, repositioning is done using 5 F, 40 cm guiding catheters (hockey stick, Cobra, etc). Insertion of the new coaxial catheter over the guidewire is facilitated by an inner flexible catheter stiffener.

Retention of device

Retention was a significant consideration for this drainage system. We decided that the use of two antimicrobial cuffs, as described prior, would be sufficient in catheter retention long term in palliative patients. A second device, a tube holding device located 5 cm prior to the entry into the skin, would aid in external retention. This location would allow for visual inspection of the site, frequent cleaning, and application of a Chlorhexidine impregnated Tegaderm. This retention device would be particularly important in the first several weeks of the device’s use to allow for subcutaneous adhesion to the antimicrobial cuffs. The exterior device was designed with the following idea: maintain catheter position, decrease the risk of infection, view signs of infection, provide comfort and be minimally obtrusive, and prevent injury. The external securement device itself would be raised slightly above the skin, as allowed with small bumpers, and be sutured in place for maximal securement. The tube of the drainage system would be tied to this device or adhered to with tape. Visualization of the insertion site is an essential aspect of this device which would also allow for the changing of bandages. This is particularly important when considering tasks of daily living. This method of securement would allow for the patient to shower and eventually bathe without too much difficulty.

Special considerations on infection

Tunneled devices have been shown to reduce complications of infection by a significant amount [17]. Antimicrobial cuffs offer additional protection and are commonly used in vascular devices. The proposal is to use two antimicrobial cuffs that would aid in preventing infection and retention of the device. The use of multiple barriers to the inner lumen, of both the sheath and the catheter, was another critical consideration. An external cap that would keep the internal components sterile was an early consideration. This would allow for sterile access to internal components. The addition of a secondary cap within the sterile region would provide a second layer of prevention of infection. Visualization of the insertion site on the catheter was also an additional important aspect we considered. Removal of the inner catheter would allow for a larger diameter than a scope, such as SpyGlass endoscopy, to pass through the channel without effort. Visual observation of the insertion site allows for assessing signs of infection or drainage and easy maintenance of the catheter.

Cost minimization

With the average cost of hospitalization in the United States reaching over $11,700 in 2020, any additional cost of the catheter and possible increased production costs of the catheter should easily be offset by the savings from the decreased hospital stay, decreased need for surgical intervention and anesthesia, and procedure cost [18]. Price adjusted for 2007, the cost of treating a single effusion with thoracentesis without inpatient hospitalization was $2,062 on average [19]. Price adjusted for 2008, the cost for a single paracentesis outpatient procedure was $1,954 [20]. Thus, the need for a cost-effective long-term solution is highly marketable.

There is scare data comparing the cost between different therapies for recurrent effusions. Olden and Holloway used base case analysis to compare the cost between Pleurx and talc pleurodesis. They found that there was similar effectiveness of quality adjusted life years (QALYs), however talc pleurodesis was less costly (talc, 0.281 QALYs and $8170.80; Pleurx, 0.276 QALYs and $9011.60) [21]. Froudarakis compared the cost of repeated thoracentesis and pleurodesis and found that repeat thoracentesis was the cheapest intervention at three-month survival where bedside pleurodesis became the cheapest at 12-month analysis (thoracentesis $4946; pleurodesis $13057) [22]. However, Froudarakis felt that indwelling pleural catheters were difficult to compare to other treatments because patient selection, techniques, and complications are different with these devices [22]. More research comparing quality adjusted life years, cost and complications is recommended for future studies.

Potential for development

To our knowledge, such a device as described above has not been developed. While we recognize the challenges of breaking into the palliative drainage catheters market, we believe there is an excellent opportunity for improvement. This catheter only requires minor modifications to existing commercially available devices; we postulate they could be efficiently manufactured without significant changes in production equipment and at a cost like what is already on the market. The largest producers of such devices are Cook Medical, Argon, Mermaid, Boston Scientific, Merit, and Teleflex, and we view these companies as the primary stakeholders for our device. These groups already have the infrastructure to manufacture a catheter with our design. Additionally, we conclude that these companies’ interests in keeping or increasing their share of the market would warrant their investment. However, we recognize the challenges of convincing these larger entities of the actual need for this design. Therefore, looking for investment from those that would use this device, such as interventional radiologists, could prove as essential secondary stakeholders in our design.

Limitations

While this catheter, like other multipurpose drainage catheters, can be used with a wide variety of pathologies, there are some instances where it may not be appropriate. This device would likely not be a good candidate for persons who fail to attend appointments. Any device which enters spaces within the body needs follow-up and care. Caution to place this device in patients with a high risk of failure to follow up is warranted. Simple paracentesis and thoracentesis procedures can be more easily monitored clinically, for complications such as pneumothoraxes, infection, or hemorrhage. While these procedures are uncomfortable and expensive, they are often monitored under direct supervision. A permanent tube may be unsightly, unsettling, and in some cases, more uncomfortable for the patient when compared to multiple procedures. Removal of the catheter with two antimicrobial cuffs would likely be difficult. While this device is intended to be long-term in the palliative population, there may be some situations where removal may be necessary, and this may be difficult due to increased adhesion. Future clinical trials are also needed to compare this novel device with other existing procedures and devices to assess its efficiency, safety, and user-friendliness.

## Conclusions

This paper describes the application of a novel device that offers definite improvements for the palliative management of chronic thoracic and abdominal effusions. Major enhancements to existing designs are an outer sleeve-sheath and an inner coaxial drainage catheter with a fluid dynamic proven tip design for efficient drainage. The outer sleeve-sheath allows for the exchange of the coaxial drainage catheter and diagnostic and therapeutic maneuvers within the thoracic and abdominal cavities. These are among the SCDC advantages over other competitive devices currently in the market.
